# HIV‐1 drug resistance among individuals who seroconverted in the ASPIRE dapivirine ring trial

**DOI:** 10.1002/jia2.25833

**Published:** 2021-11-11

**Authors:** Urvi M. Parikh, Kerri J. Penrose, Amy L. Heaps, Elias K. Halvas, B. Jay Goetz, Kelley C. Gordon, Russell Hardesty, Rahil Sethi, William Schwarzmann, Daniel W. Szydlo, Marla J. Husnik, Uma Chandran, Thesla Palanee‐Phillips, Jared M. Baeten, John W. Mellors, the MTN‐020 Study Team

**Affiliations:** ^1^ Department of Medicine, Division of Infectious Diseases University of Pittsburgh Pittsburgh Pennsylvania USA; ^2^ Fred Hutchinson Cancer Research Center Seattle Washington USA; ^3^ Wits Reproductive Health and HIV Institute Johannesburg South Africa; ^4^ Departments of Global Health Medicine Epidemiology University of Washington Seattle Washington USA; ^5^ Present address: Albany Stratton Veterans Administration Medical Center Albany NY USA; ^6^ Present address: Gilead Sciences Foster City CA USA

**Keywords:** dapivirine, HIV‐1 drug resistance, HIV‐1 prevention, next‐generation sequencing, non‐nucleoside reverse transcriptase inhibitors (NNRTI), pre‐exposure prophylaxis (PrEP)

## Abstract

**Introduction:**

A potential concern with the use of dapivirine (DPV) for HIV prevention is the selection of a drug‐resistant virus that could spread and reduce the effectiveness of non‐nucleoside reverse transcriptase (NNRTI)‐based first‐line antiretroviral therapy. We evaluated HIV‐1 seroconversions in MTN‐020/ASPIRE for selection of drug resistance and evaluated the genetic basis for observed reductions in susceptibility to DPV.

**Methods:**

MTN‐020/ASPIRE was a placebo‐controlled, Phase III safety and effectiveness study of DPV ring for HIV‐1 prevention conducted at 15 sites in South Africa, Zimbabwe, Malawi and Uganda between 2012 and 2015. Plasma from individuals who seroconverted in ASPIRE was analysed for HIV‐1 drug resistance using both population Sanger sequencing and next‐generation sequencing (NGS) with unique molecular identifiers to report mutations at ≥1% frequency. DPV susceptibility of plasma‐derived recombinant HIV‐1 containing bulk‐cloned full‐length reverse transcriptase sequences from MTN‐020/ASPIRE seroconversions was determined in TZM‐bl cells. Statistical significance was calculated using the Fisher's exact test.

**Results:**

Plasma from all 168 HIV seroconversions were successfully tested by Sanger sequencing; 57 of 71 DPV arm and 82 of 97 placebo (PLB) arm participants had NGS results at 1% sensitivity. Overall, 18/168 (11%) had NNRTI mutations including K101E, K103N/S, V106M, V108I, E138A/G, V179D/I/T and H221Y. Five samples from both arms had low‐frequency NNRTI mutations that were not detected by Sanger sequencing. The frequency of NNRTI mutations from the DPV arm (11%) was not different from the PLB arm (10%; *p* = 0.80). The E138A mutation was detected in both the DPV (3 of 71 [4.2%]) and PLB arm (5 of 97 [5.2%]) and conferred modest reductions in DPV susceptibility in some reverse transcriptase backgrounds but not others.

**Conclusions:**

HIV‐1 drug resistance including NNRTI resistance did not differ between the DPV and placebo arms of the MTN‐020/ASPIRE study, indicating that drug resistance was not preferentially acquired or selected by the DPV ring and that the preventive benefit of DPV ring outweighs resistance risk.

## 1 INTRODUCTION

Women in Sub‐Saharan Africa have a disproportionate risk of HIV infection [1], highlighting the pressing need for safe and effective prevention strategies that allow for agency and choice. Dapivirine (DPV) is a potent diarylpyrimidine (DAPY) non‐nucleoside reverse transcriptase inhibitor (NNRTI). A silicone elastomer intravaginal matrix ring containing 25 mg of DPV (DVR) used monthly is recommended by the World Health Organization (WHO) as part of combination prevention in women [2]. DVR has received a positive opinion from the European Medicines Agency under the Article 58 procedure. Two Phase III HIV prevention trials, Microbicide Trials Network (MTN)‐020/A Study to Prevent Infection with a Ring for Extended Use (ASPIRE) and International Partnership for Microbicides (IPM) 027/The Ring Study, showed a reduction in the risk of HIV‐1 infection among African women by 27% and 35%, respectively, and suggested greater protection correlated with higher adherence [3, 4, 5]. Data from the open‐label extension study MTN‐025/HOPE showed higher adherence and indicated lower incidence than in ASPIRE, based on modelling [6].

A potential concern with the use of DVR is that the selection of resistant virus with breakthrough infection and its subsequent spread could reduce the effectiveness of NNRTI‐based first‐line antiretroviral therapy (ART). Plasma concentrations of DPV in women using the ring are low but detectable, such that resistant virus could be selected systemically [3, 4]. In vitro, DPV selects for common NNRTI mutations at HIV‐1 reverse transcriptase (RT) positions 90, 100, 101, 106, 138, 179, and 181 [7] but data on resistance selection in vivo are limited because DPV is not used therapeutically. It was thus critical to use sensitive and accurate methods to evaluate HIV‐1 seroconversions in ASPIRE.

National survey data from the WHO shows significant increases in pre‐treatment drug resistance in the past five years, with prevalence rates between 10% and 30% in multiple countries in Sub‐Saharan Africa [8, 9]. A total of 91% of HIV‐1 clones derived from individuals from South Africa on failing ART had cross‐resistance to DPV, ranging from 3‐fold to greater than 500‐fold decrease in susceptibility compared to wild‐type HIV‐1 [10]. In vitro studies found the L100I/K103N combination to confer the highest level of cross‐resistance to DPV [11]. Also concerning is the high prevalence of the polymorphism E138A, which occurs naturally in 5% of treatment‐naïve HIV‐1‐subtype C‐positive individuals and is selected by other DAPY‐class NNRTIs causing 3‐fold resistance to etravirine and rilpivirine [12, 13]. The impact of circulating resistance strains or the E138A mutation on DVR protective efficacy is not known.

We previously described select resistance mutations identified from Sanger sequencing in individuals who seroconverted in ASPIRE [3]. The current study reports new data on full‐length protease and RT mutation frequency detection by Sanger sequencing, evaluates the frequency of minor variant detection by sensitive next‐generation sequencing, and systematically analyses DPV susceptibility of HIV‐1 with NNRTI mutations, to provide the first comprehensive understanding of resistance in individuals who acquired HIV‐1 while using DPV or placebo rings in ASPIRE.

## 2 METHODS

ASPIRE was a Phase III double‐blinded placebo‐control randomized trial of DVR conducted between August 2012 and June 2015 at 15 clinical research sites in Malawi, Uganda, South Africa, and Zimbabwe, as described previously [3]. The trial protocol (available at mtnstopshiv.org) was approved by the ethics committee/review board at each site (as detailed in **Table** **S1**) and all participants provided written informed consent. Briefly, 2629 sexually active HIV‐negative women aged 18–45 were randomly assigned (1:1) to receive a monthly DPV or placebo ring. Participants were tested monthly for HIV seroconversion using two concurrent rapid tests, and positive results were confirmed by Western blot and HIV‐1 RNA PCR (Abbott M2000 or Roche TaqMan). Plasma specimens were stored quarterly and when HIV‐1 seroconversion was first detected. The current study was conducted using plasma samples obtained at the visit where HIV‐1 seroconversion was detected and when the investigational product was withdrawn. The present analysis includes all 168 participants who seroconverted in ASPIRE, plus an additional three who enrolled into ASPIRE with undetected acute infection, and three who seroconverted after cessation of product use, but before exiting the study.

### 2.1 Sanger sequencing

We performed Sanger sequencing of HIV‐1 protease (amino acids 1–99) and full‐length RT (amino acids 1–560) using an in‐house assay with primers optimized for non‐B HIV‐1 subtypes. RNA was extracted from plasma by guanidinium thiocyanate lysis/isopropanol precipitation and was amplified with the SuperScript^TM^ III One‐Step RT PCR System containing Platinum^TM^ Taq DNA polymerase (Life Technologies) using a final concentration of 200 μM primers targeting HIV‐1 protease and RT (OF‐1 5’‐GAGGGACACCAAATGAAAGAYTG‐3’ and 3908‐ 5’‐CACAGCTGGCTACTATTTCTTTTGC‐3’) in the following thermocycling protocol: 1 hour at 50°C; 2 minutes at 94°C followed by 40 cycles of (15 seconds at 94°C, 30 seconds at 50°C, 2.5 minutes at 68°C) and final extension at 65°C for 5 minutes. Reactions were visualized on a 1% agarose gel and excess primers and nucleotides were hydrolysed with ExoSAP‐It (Affymetrix). Cycle sequencing (Applied Biosystems™ 3130xL) was performed using the BigDye™ Terminator v3.1 Cycle Sequencing kit (Applied Biosystems™) using eight bidirectional primers spanning the protease and RT gene regions. Electropherograms were evaluated for quality and mixture presence (20% threshold) using Sequencher^TM^ DNA Analysis Software (v5.4, Gene Codes Corporation). Resistance mutations and viral subtypes were identified using the Stanford Genotypic Resistance Interpretation Algorithm v9.0 [14].

### 2.2 Next‐generation sequencing (NGS)

Modified from Boltz et al., NGS libraries were prepared using PCR and sticky‐end linker ligation to tag individual cDNA molecules with unique molecular identifiers (UMIs) and amplify HIV‐1 templates for sequencing on the Illumina® Miseq® platform [15]. RNA was extracted from plasma by guanidinium thiocyanate lysis/isopropanol precipitation and was reverse transcribed using Superscript III (Invitrogen) with 30 nM primers that contained 10 degenerate nucleotides to serve as UMIs for each template (5’‐GTATCGAAGTCATCCTGCTAGNNNNNNNNNNTTCTTGTCT GGTGTGGTAAATCC‐3’). Reactions were treated with Exonuclease I (NEB) and Shrimp Alkaline Phosphatase (NEB) followed by RNase H (NEB) treatment. cDNA was precipitated overnight and HIV‐1 RT was amplified in five reactions using Kapa HiFi Hot Start Uracil (Kapa Biosystems) and 300 nM dU‐containing primers (forward 5’‐AAACAAUGGCCAUTG ACAGAAGA‐3’; reverse 5’‐GGUAUCGAAGUCAUCCUGCTAG‐3’) using the following cycling conditions: 3 minutes at 95°C for 1 cycle; then 20 seconds at 95°C, 30 seconds at 60°C, 2 minutes at 68°C with 5 seconds added onto each subsequent cycle for 10 cycles. Replicate wells were combined and PCR product was purified with KAPA Pure Beads (Kapa Biosystems) at a 0.7 ratio of beads to the product. A second round of PCR in five replicate wells using Kapa HiFi Hot Start Uracil and 300 nM dU‐containing primers (forward 5’‐ GUGGAGAAAAUUAGTAGATTTCAGGGARC ‐3’; reverse 5’‐ GGUAUCGAAGUCAUCCUGCTAG ‐3’) was performed using similar cycling conditions with the elongation phase repeating for 35 cycles. Replicate wells were combined and PCR product was re‐purified with KAPA Pure Beads (0.7 ratio beads:product). PCR products were prepared for ligation of Illumina adaptors by treatment with uracil DNA glycosylase (NEB), cleavage, and removal of DNA at abasic sites by denaturation with 2N NaOH, which created 3’ overhangs for efficient ligation. Reactions were neutralized with 2M Trizma hydrochloride. DNA was renatured and precipitated overnight. Ligation reactions were set up overnight at 25°C with Illumina adaptors at equimolar concentrations. Adaptor‐ligated libraries were purified using the Blue Pippin DNA size selection system (Sage Science) and quantified with the KAPA Library Quantification Kit (Kapa Biosystems). Samples were normalized to 4 nM and pooled prior to sequencing on the Illumina MiSeq at a 12 pM final concentration.

### 2.3 Bioinformatics

Bioinformatics analysis was performed using an in‐house developed pipeline. FASTQC v.0.11.7 was run to test for read quality prior to concatenating R1 and R2 paired ends. FASTX toolkit v0.0.13 was used to remove reads with quality <PHRED 20 and convert FASTQ to FASTA. Samples were then de‐multiplexed and binned based on index pair usage. Sequences were aligned to a subtype C reference using BLAST v2.6.0 and consensus sequences were made for each UMI Index ID with the following rules: reads were included if they followed the Zhou model [16] and 80% homology was used to call bases while allowing 1 mismatch base per consensus sequence. For 95% confidence, 298 and 54 UMI consensus sequences were required to call 1% and 5% minor variants, respectively. Maximal sensitivity was determined on a per sample basis depending on the number of consensus sequences per sample. Ratios of mutations were determined using HXB2 as a wild‐type comparator. Cross‐sample contamination was detected and removed with a phylogenetic analysis of sample virions using Phylip v3.697.

### 2.4 Generation of recombinant HIV‐1 containing full‐length plasma‐derived HIV‐1 reverse transcriptase

Infectious virus containing plasma‐derived RT was generated as previously described [10]. Briefly, using the In‐Fusion® HD Cloning System (Clonetech), plasma‐derived full‐length RT was bulk cloned into HIV‐1_LAI_ [17] using silent restriction sites Bcl1 and Xho1 to preserve sequence diversity, and the resultant plasmid DNA was purified using the PureYield™ Plasmid Midiprep System (Promega). Lipofectamine2000 (Life Technologies) transfection into 293T cells was performed to generate infectious viral clones.

### 2.5 Mutant reversions

Single colonies containing E138A were isolated from bulk‐cloned plasma‐derived plasmid preparations. QuickChange Lightning Multisite or QuickChange II XL (Agilent) site‐directed mutagenesis kits were used to sequentially revert E138A and other NNRTI mutations to wild type. Plasmid DNA from individual colonies was isolated using QIAprep spin miniprep kit (Qiagen) and sequenced using Sanger sequencing. Plasmid DNA was isolated from large‐scale colony preps using the Pureyield midiprep system (Promega). These plasmids were used to generate viral stocks as described above and the sequence was again confirmed from RNA isolated from the viral stocks.

### 2.6 HIV‐1 phenotyping

A normalized input of 300 relative light units (RLU) was used to infect untreated or antiretroviral (ARV)‐treated TZM‐bl cells in a luciferase‐based single cycle drug susceptibility assay (Britelite Plus; Perkin‐Elmer) as previously described [18]. DPV was kindly provided by International Partnership for Microbicides (Silver Spring, MD). Nevirapine (NVP), efavirenz (EFV), etravirine (ETR), and rilpivirine (RPV) were obtained through the AIDS Research and Reference Reagent Program, Division of AIDS, NIAID, NIH.

### 2.7 Statistical analysis

Novel resistance mutations using HIV‐1_HXB2_ as the reference were sought using the Hellinger distance of amino acid distributions across each codon and compared against a permuted null created with 1000 iterations. Amino acid differences were considered to be significant if the Holm‐Bonferroni FWER‐adjusted *p*‐value was <0.05. Four‐parameter, non‐linear regression for curve fitting was used to generate IC_50_ values using GraphPad Prism 6 software (GraphPad Software, Inc). Fold‐change was calculated as IC_50_ of mutant HIV‐1/wild‐type HIV‐1 and *p*‐values were calculated using Linear mixed‐effects models and used with Satterthwaite approximations to determine significance, with Bonferroni corrections for multiple comparisons. Phylogenetic analysis using MEGA7 [19] was done to ensure that plasma‐derived cloned virus sequence clustered with the virus in the original plasma sample. Fisher's exact test was used to compare the occurrence of NNRTI mutations between the two arms.

## 3 RESULTS

### 3.1 Proportion of HIV‐1 seroconversions with drug resistance, by randomization arm, using Sanger sequencing

**Figure 1:**
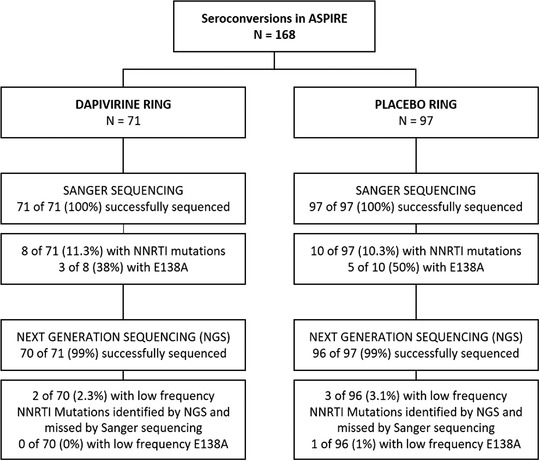
Consort diagram.

Plasma samples, obtained at the visit at which HIV‐1 seroconversion was detected and at which time study medication was withdrawn, were successfully tested by Sanger sequencing for all 168 participants who seroconverted in ASPIRE, including 71 participants from the DVR arm, and 97 participants from the placebo ring arm (Figure 1). Of the samples tested, the median (range) of plasma HIV‐1 RNA in the placebo arm was 150,173 (89 to >10,000,000) copies/ml and in the DPV arm was 137,471 (61 to >10,000,000) copies/ml. Of the 168 seroconversions, 10 of 97 (10.3%) in the placebo arm and 8 of 71 (11.3%) in the DPV arm had NNRTI resistant‐HIV‐1. The overall occurrence of NNRTI mutations was not different by arm (Fisher's exact test: *p*‐value 0.80). Major NNRTI mutations detected included K101E, K103N, K103S, V106M, V108I, E138A/G, V179D/I/T and H221Y, but the frequency of detection of each of these mutations did not differ by arm (Table 1). The majority of participants were infected with subtype C HIV‐1 (*n* = 155) while six with subtype A, three with subtype D, and one each with AE/A, B, C/F and C/K. Amino acid changes, including G335D, N348I, T369I, A371V, and A376S [20, 21] associated with DPV or NNRTI resistance were not detected in the connection or RNAse H domains of HIV‐1 RT. Two samples had nucleoside/tide reverse transcriptase inhibitor (NRTI) mutations; one with E44D and a second with A62A/V with the NNRTI mutation H221Y. One sample had the L90M protease inhibitor resistance mutation with no other NRTI or NNRTI mutations.

**Table 1:** Frequency of non‐nucleoside reverse transcriptase inhibitor mutations by Sanger sequencing among participants in ASPIRE who acquired HIV‐1 infection after enrolment while on study product, by the arm

NNRTI mutation^a^	Placebo (PLB) ring *N* = 97	Dapivirine (DPV) ring *N* = 71	*p*‐Value^b^
K101E	1 (1.0%)	1 (1.4%)	1.00
K103N	1 (1.0%)	2 (2.8%)	0.57
K103S	0 (0%)	1 (1.4%)	0.42
V106M	0 (0%)	1 (1.4%)	0.42
V108I	0 (0%)	1 (1.4%)	0.42
E138A	5 (5.2%)	3 (4.2%)	1.00
E138G	0 (0%)	1 (1.4%)	0.42
V179D	2 (2.1%)	1 (1.4%)	1.00
V179I/T	0 (0%)	1 (1.4%)	0.42
H221Y	1 (1.0%)	1 (1.4%)	1.00

^a^ Non‐nucleoside reverse transcriptase mutations listed in the table were identified by the Stanford Genotypic Resistance Interpretation Algorithm v9.0 [14]. Additional polymorphic/accessory mutations were detected as follows: V90I (1 PLB ring [1%]; 2 DPV ring [2.8%; *p* = 0.57]), and K103R (3 DPV ring only [4.2%; *p* = 0.074]. No cases of the following non‐nucleoside reverse transcriptase inhibitor (NNRTI)‐associated mutations occurred in this cohort: A98G, K101H/P/Q/R/N, L100I, K103H/T/Q/E, V106A/I/T, I132M/L, E138K/Q/R, V179E/F/L/T, Y181C/I/V, Y188C/H/L, G190A/E/Q/S, P225H, F227C/I/L/V, M230I/L, Y232H, L234I, P236L, K238T/N, Y318F, or N348I.

^b^ Calculated using Fisher's exact test for non‐zero values.

In addition to the 168 seroconversions on the study product, Sanger sequencing was performed on 3 of 3 participants who were acutely infected at enrolment, and 3 of 3 participants who seroconverted after their cessation of study product. Of these six, only one participant from the placebo arm had an NNRTI resistance mutation detected (K103N) in a sample collected 8 weeks after cessation of the placebo ring.

### 3.2 Identification of low‐frequency mutants by next‐generation sequencing

Plasma UMI‐NGS was successfully completed for 166 of 168 seroconversions. Two samples, one from each arm, did not amplify despite having sufficient viral RNA (Figure 1). The majority of samples (139 of 166) had mutation detection sensitivity of 1% (based on ≥ 298 UMIs). All mutations detected by Sanger sequencing were confirmed by UMI‐NGS. Only five samples were identified that had low‐frequency NNRTI‐associated polymorphisms or mutations not previously detected by Sanger sequencing, with the mutant frequency indicated in parentheses: K101R (1.4%), V108I (1.7%), E138A (9.3%), V179G (1.0%) and Y181C (1.2%). The detection of low‐frequency mutations was evenly distributed across study arms (Table 2). The overall frequency of mutations and the frequency of specific mutations detected by NGS was not different between arms. No novel mutations were associated with seroconversion during dapivirine ring use compared to placebo ring use.

**Table 2:** Frequency of minor variant non‐nucleoside reverse transcriptase inhibitor mutations among participants in ASPIRE who acquired HIV‐1 infection after enrolment while on study product, by the arm

Sensitivity of drug resistance mutation detection	No. of unique molecular identifiers (UMI) needed*	No. of DPV ring samples (UMI range)	No. of placebo ring samples (UMI range)	Minor variant NNRTI mutations (% detected) by PID and study arm^a^
1%	298	57 (410–25,311)	82 (407–27,797)	K101R (1.4%) PID 1 (PLB) V108I (1.7%) PID 2 (DPV) E138A (9.3%) PID 3 (PLB) V179G (1.0%) PID 4 (DPV) Y181C (1.2%) PID 5 (PLB)
5%	58	7 (71–225)	7 (77–266)	None
10%	28	1 (41)	4 (28–43)	None
20%	13	1 (27)	1 (20)	None
>20%^b^	<13	4 (2–6)	2 (2–11)	Indeterminate

Abbreviations: NNRTI, non‐nucleoside reverse transcriptase inhibitor; PID, participant identification; PLB, placebo ring arm; DPV, dapivirine ring arm.

^a^ Minor variant is defined as mutations present at or below 20% frequency in the sample.

^b^ Six samples were tested by next‐generation sequencing, but did not yield a sufficient number of unique molecular identifiers to detect minor variants.

* With a 95% probability of detection at specific frequencies.

### 3.3 Susceptibility to dapivirine of plasma‐derived HIV‐1 with NNRTI mutations

We determined DPV susceptibility of plasma‐derived recombinant HIV‐1 from the eight participants in the DPV ring arm, and the 10 participants in the PLB ring arm that had HIV‐1 with NNRTI mutations (Table 3). Single NNRTI mutations (with the exception of two PLB participants with E138A) did not reduce susceptibility to DPV (DPV‐8, PLB 4–10), with FC ranging from 0.4 to 1.5. The two PLB participants with E138A alone had 3.0 and 4.2‐FC reduction in DPV susceptibility (*p* < 0.001). HIV‐1 with dual NNRTI mutations (DPV‐1‐7 and PLB‐1) had the greatest reduction in susceptibility to DPV, with FC ranging from 6 to 22 (Table 3).

**Table 3:** Susceptibility to dapivirine of plasma‐derived viruses from ASPIRE seroconverters infected with HIV‐1

Study arm	Participant ID	Major NNRTI mutations	Subtype	HIV‐1 RNA (copies/ml)	Dapivirine IC_50_ ±SD* (nM)	Fold‐change^a^	*p*‐Value^b^	Plasma dapivirine levels (pg/ml)^c^
Dapivirine	DPV‐WT	–			0.7 ± 0.2	–		206
	DPV‐1	E138A, V179I/T	C	>10,000,000	4.0 ± 0.8	5.7	<0.001	182
	DPV‐2	V108I/V, E138A	C	159,273	1.4 ± 0.3	2.0	<0.001	73.6
	DPV‐3	E138A, V179D	C	9752	0.4 ± 0.1	0.57	0.79	508
	DPV‐4	K101E, E138G	C	771,412	4.3 ± 0.4	6.1	<0.001	477
	DPV‐5	K103S, V106M	C	4854	8.2 ± 0.5	12	<0.001	179
	DPV‐6	V90I, K103N	C	2109	4.5 ± 0.4	6.4	<0.001	209
	DPV‐7	V90I, K103N	C	7287	15.3 ± 2.4	22	<0.001	60
	DPV‐8	H221Y	C	142,690	0.4 ± 0.1	0.57	0.68	334
Placebo	PLB‐WT	‐			0.9 ± 0.4	–		0
	PLB‐1	K101E, E138A	C	22,876	4.6 ± 1.4	4.6	<0.001	0
	PLB‐2	E138A	C	579,843	4.2 ± 0.9	4.2	<0.001	0
	PLB‐3	E138A	C	92,967	3.0 ± 0.8	3.0	<0.001	0
	PLB‐4	E138A	C/F	199,934	1.2 ± 0.1	1.2	1.0	0
	PLB‐5	E138A	C	7,969,414	1.4 ± 0.3	1.4	1.0	0
	PLB‐6	V109I	C	209,566	0.8 ± 0.1	0.9	1.0	0
	PLB‐7	K103N	C	1,150,214	1.4 ± 0.3	1.5	1.0	0
	PLB‐8	V179D	C	23,608	1.1 ± 0.1	1.2	1.0	0
	PLB‐9	V179D	C	468,105	0.4 ± 0.1	0.57	0.92	0
	PLB‐10	H221Y^d^	C	70,420	0.4 ± 0.02	0.4	1.0	0

* Plasma‐derived recombinant virus from ASPIRE participants infected with wild‐type (WT) HIV‐1 with no mutations in the reverse transcriptase gene were used as the dapivirine (DPV) arm and placebo (PLB) arm WT controls. The IC_50_ is a composite from 8 placebo‐arm participants and 5 DPV‐arm participants, respectively, each tested in three independent experiments. IC_50_ values were generated for each plasma‐derived recombinant virus with non‐nucleoside reverse transcriptase inhibitor (NNRTI) resistance mutations in three to five independent experiments.

^a^ Calculated using the IC_50_ values from DPV‐WT as the denominator for DPV arm viruses, and PLB‐WT for PLB arm viruses.

^b^ *p*‐values with Bonferroni corrections for multiple comparisons.

^c^ Plasma DPV levels were measured from the same blood draw collected for phenotypic testing. Plasma DPV levels of ≥95 pg/ml indicate some level of adherence. The average DPV level is shown for the composite DPV‐WT.

^d^ PLB‐10 also had the nucleoside reverse transcriptase inhibitor mutation A62AV.

### 3.4 Systematic evaluation of the relative contribution of E138A and other NNRTI mutations to dapivirine susceptibility

The RT polymorphism E138A occurs naturally in 5% of treatment‐naïve HIV‐1‐subtype C‐positive individuals, but can also be selected by the DAPY class of NNRTIs [13, 22]. E138A was the most frequently detected mutation in HIV‐positive ASPIRE participants. The frequency was not different between arms (4.2% DPV vs. 5.2% PLB; *p* = 1.00) in ASPIRE (Table 1) but E138A was detected more frequently in DVR seroconversions in the Ring Study [4]. Due to concerns that E138A could reduce the protective efficacy of DVR, we phenotyped clonal recombinant isolates from two samples from each arm (DPV‐1, DPV‐2, PLB‐1 and PLB‐2) to further investigate DPV susceptibility. We also systematically reverted each NNRTI mutation to wild‐type and phenotyped those clones to determine the relative effects of E138A alone and in combination with other NNRTI mutations compared to a bulk‐cloned wild‐type control.

The E138A/V179I clone (DPV‐1) conferred the greatest reduction in DPV susceptibility (19‐FC), followed by the E138A/V179T clone (11‐FC). V179I or V179T alone was susceptible to DPV (1.0 and 1.4‐FC), while E138A alone had 4.7‐FC with respect to wild‐type HIV‐1. Similarly, the HIV‐1 V108I/E138A clone (DPV‐2) had a greater reduction in DPV susceptibility (3.9‐FC) than HIV‐1 with V108I or E138A alone (0.8 and 2.6‐FC). The K101E/E138A clone (PLB‐1) conferred 12‐FC reduction in DPV susceptibility, which is greater than either mutation alone (K101E, 3.6‐FC; E138A, 4.2‐FC). The IC_50_ of the single colony purified and bulk‐cloned HIV‐1 recombinants were equivalent for the second placebo arm samples (PLB‐2). Reverting all mutations to wild‐type restored susceptibility to DPV for all samples (Table 4).

**Table 4:** Systematic evaluation of the relative contributions of E138A and other NNRTI mutations to dapivirine susceptibility

Recombinant	Description	NNRTI genotype	Mean dapivirine IC_50_ ± SD* (nM)	Fold‐change from bulk‐cloned WT^a^	*p*‐Value^c^
**DPV‐1**	Bulk‐cloned	E138A, V179IT	4.0 ± 0.83	6.1	<0.01
	Single colony	E138A, V179I	12.5 ± 3.85	19	<0.01
	Single colony	E138A, V179T	7.39 ± 2.63	11	<0.01
	V179IT revertant	E138A	3.10 ± 0.82	4.7	<0.01
	E138A/V179T revertant	V179I	0.67 ± 0.13	1.0	ns
	E138A/V179I revertant	V179T	0.92 ± 0.26	1.4	ns
	E138A/V179IT revertant	Wild type	0.75 ± 0.11	1.1	ns
**DPV‐2**	Bulk‐cloned	V108I/V, E138A	1.43 ± 0.36	2.2	<0.01
	Single colony	V108I, E138A	2.58 ± 0.86	3.9	<0.01
	E138A revertant	V108I	0.53 ± 0.003	0.8	ns
	V108I revertant	E138A	1.74 ± 0.49	2.6	<0.01
	V108I/E138A revertant	Wild type	0.50 ± 0.10	0.8	ns
**PLB‐1**	Bulk‐cloned	K101E, E138A	4.60 ± 1.44	4.7	<0.01
	Single colony	K101E, E138A	11.7 ± 1.37	12	<0.01
	E138A revertant	K101E	3.54 ± 0.64	3.6	<0.01
	K101E revertant	E138A	4.09 ± 1.12	4.2	<0.01
	K101E/E13A revertant	Wild type	0.96 ± 0.10	1.0	ns
**PLB‐2**	Bulk‐cloned	E138A	4.19 ± 0.89	4.3	<0.01
	Single colony	E138A	4.59 ± 1.67	4.7	<0.01
	E138A revertant	Wild type	0.89 ± 0.06	0.9	ns

* 50% in vitro dapivirine concentration (IC_50_) mean and standard deviation (SD) calculated from three to six independent determinations.

^a^ Calculated using the IC_50_ value from the bulk‐cloned wild‐type virus that was run concurrently to the reversion samples. The denominator to calculate fold‐change for the dapivirine arm samples was 0.66 nM and for the placebo arm samples was 0.97 nM. Values were also calculated using a single colony WT and reversion wild‐type and FC was comparable (data not shown).

^c^ *p*‐Values with Bonferroni corrections for multiple comparisons; ns, not significant.

**Figure 2:**
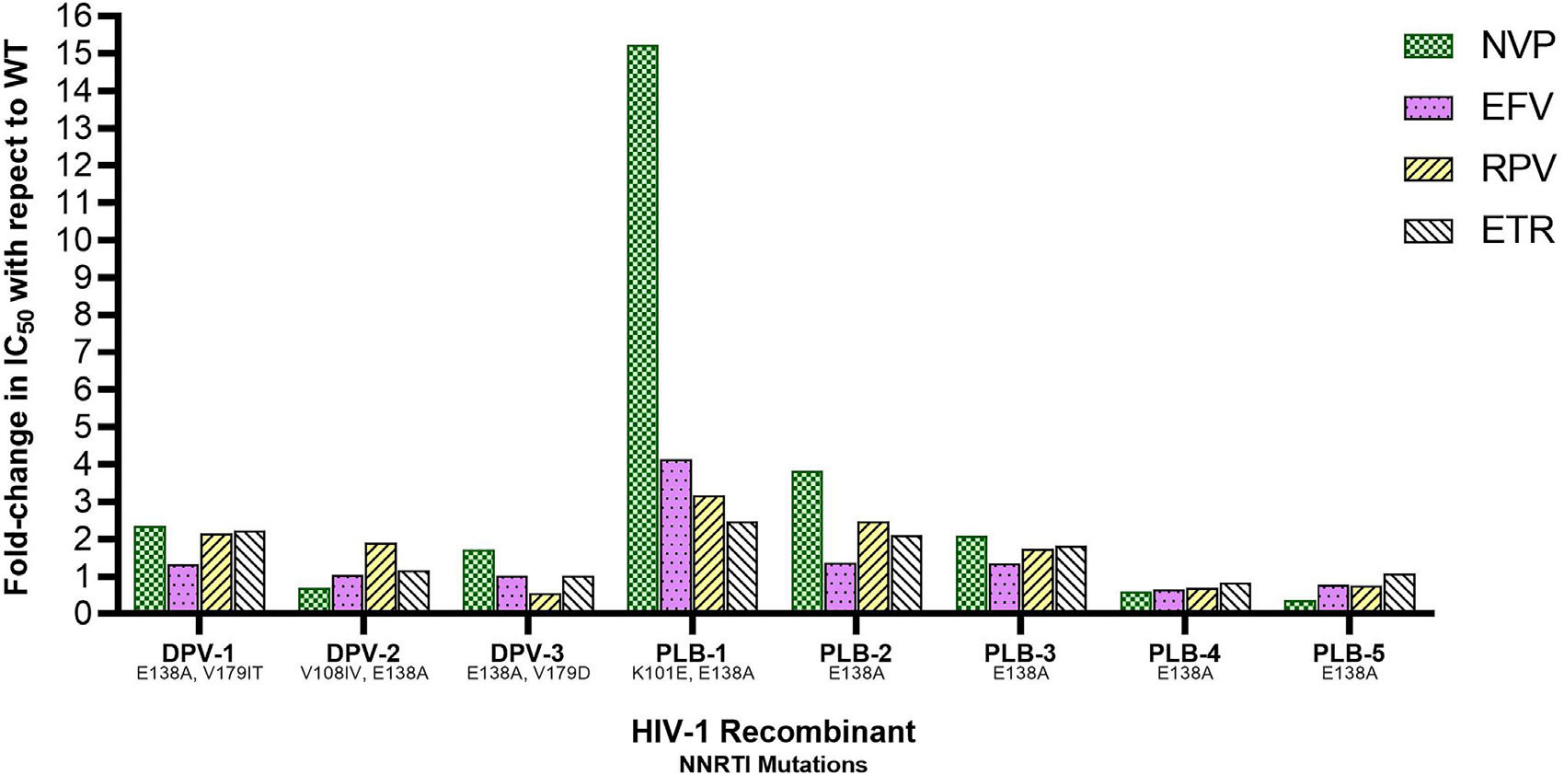
Cross‐resistance of plasma‐derived HIV‐1 with E138A to non‐nucleoside reverse transcriptase inhibitors used for antiretroviral therapy. Fold‐change in 50% in vitro concentration (IC_50_) of the non‐nucleoside reverse transcriptase inhibitors nevirapine (NVP, checkered bar), efavirenz (dotted bar), rilpivirine (RPV, backward diagonal bar) and etravirine (forward diagonal bar) with respect to wild‐type HIV‐1 as determined in TZM‐bl cells is shown for three recombinant plasma‐derived HIV‐1 from participants in the dapivirine (DPV) arm of ASPIRE. These recombinants are depicted as follows: DPV‐1 is HIV‐1_E138A/V179IT_, DPV‐2 is HIV‐1_V108IV/E138A_, DPV‐3 is HIV‐1_E138A/V179D_. Fold‐change IC_50_ is also shown for 5 placebo (PLB) arm plasma‐derived HIV‐1 as follows: PLB‐1 is HIV‐1_K101E/E138A_, PLB‐2_E138A_, PLB‐3_E138A_, PLB‐4_E138A_, PLB‐5_E138A_.

All E138A‐containing bulk‐cloned viruses were tested for cross‐resistance to commonly used NNRTIs for ART, including nevirapine, efavirenz, rilpivirine and etravirine. Except for PLB‐1, which had a moderate level of cross‐resistance to nevirapine (15‐fold), other virus/inhibitor combinations yielded low (<5‐fold) to no cross‐resistance (Figure 2).

## 4 DISCUSSION

In both ASPIRE and the Ring Study, DVR showed modest efficacy in reducing HIV risk in women. The current comprehensive analysis of drug resistance among seroconversions in the ASPIRE trial reassuringly revealed no difference in NNRTI resistance in women who seroconverted while using DPV or placebo ring.

Using Sanger sequencing, the frequency of NNRTI resistance in seroconversions from ASPIRE was high (11%), but consistent with the prevalence of pre‐treatment NNRTI resistance in Sub‐Saharan Africa as reported by recent surveys [23, 24, 25]. We were unable to determine whether DPV levels in the genital tract achieved with continual monthly DPV ring use was able to block infection by NNRTI‐resistant HIV‐1. DPV is cleared from cervicovaginal fluid within one week of ring removal, and from cervical tissue, within 3days of ring removal [26, 27]. The greatest risk of breakthrough infection with NNRTI‐resistant virus may occur in women who remove the dapivirine ring for several days before replacing it, which may result in low‐but‐detectable DPV levels at the time of HIV exposure. In a study of ring safety in adolescents (age 15–17), 91% of expulsions and removals were reported to be less than 12 hours in duration [28]. In simulated real‐world conditions, the recovery of DPV was not affected by ring exposure to common substances including bleach, personal lubricants, detergents and bath salts. Daily release of DPV was also not modified with intermittent ring use; however, due to the strong correlation of ring use with protection, there could be a risk of breakthrough infection with or without resistance in individuals who use the ring inconsistently [29, 30].

Importantly, the resistance rate seen in this analysis did not differ by study arm, indicating that resistance was likely transmitted and not selected by DPV ring use; by Sanger sequencing, 11.3% of seroconversions had NNRTI resistance in the DVR arm while 10.3% of seroconversions had NNRTI resistance in the PLB arm. Full‐length RT sequences were also evaluated because C‐terminal domain mutations have been associated with decreased susceptibility to other DAPY class inhibitors like etravirine [20, 31]. In the ASPIRE dataset, novel amino acid changes in the RT, RNAse H or the connection domains were not found to be associated with DPV ring by Sanger sequencing analysis.

A novel and sensitive UMI‐NGS method was used to identify low‐frequency mutations in HIV‐1 from participants who seroconverted in ASPIRE. The use of UMIs allowed the determination of template sampling depth and a per sample evaluation of resistance detection sensitivity, as well as post‐sequencing correction of PCR bias and sequencing error through bioinformatics tools [16]. The majority of samples (84%) had a detection limit of 1% mutation frequency. The use of UMIs is required for specificity of drug resistance calling under 5% [15, 32, 33]. NGS of HIV‐1 in plasma samples at seroconversion in ASPIRE showed no significant difference in NNRTI drug resistance mutation frequency between the DPV and PLB arms (2.3% DPV ring versus 3.1% PLB ring seroconversions with low‐frequency mutants, *p* = 0.76). Only five new NNRTI mutations were detected at frequencies below 20% and these were equally distributed by arm. One additional detection of E138A at 9.3% mutant frequency occurred in the PLB arm that was not detected by Sanger sequencing. These data confirm the Sanger sequencing results and suggest that NNRTI‐resistant HIV was not selected by DPV ring use. The analysis of seroconversions from the Ring Study had similar findings to the current study, with no difference in resistance frequency between study arms [34].

The naturally occurring polymorphism E138A was closely evaluated because of concerns of its potential association with DPV resistance. By evaluating plasma‐derived bulk‐cloned virus, then separating individual genotypes and sequentially reverting them to wild‐type, an exhaustive assessment of the role of the E138A mutation in dapivirine resistance was completed. HIV‐1 with E138A was associated with modest reductions in DPV susceptibility in some RT backgrounds, but not others. The frequency and extent of reduced susceptibility associated with E138A as the major variant was independent of the ASPIRE study arm. These phenotypic data provide reassurance that the E138A mutation was not selected by the DPV ring and may not be likely to reduce the efficacy of the DPV ring for HIV‐1 prevention.

Reduced susceptibility to DPV ranging from 6‐ to 22‐fold was noted with some genotypes with major NNRTI mutations (Table 3), which is consistent with cross‐resistance observed among HIV‐1 subtype C isolates from individuals experiencing failure of first‐line NNRTI‐based ART [10]. Continued evaluation of NNRTI resistance patterns that emerge in individuals who seroconvert while using DPV ring post‐licensure will be important in the context of rising pre‐treatment NNRTI resistance rates in countries planning DPV ring rollout [9, 35, 36].

## 5 CONCLUSIONS

In conclusion, genotyping and phenotyping analysis of seroconversions in the ASPIRE study found that DPV ring use was not associated with the selection of NNRTI resistance. Thus, the preventive benefit of the DPV ring outweighs the drug resistance risk. Monitoring of drug resistance should be considered an important component of DPV ring rollout to ensure the DPV ring remains effective in communities with high rates of transmitted NNRTI resistance and to maintain antiretroviral options for both HIV‐1 treatment and prevention in the future.

## COMPETING INTERESTS

U.M.P, K.J.P, A.L.H, E.K.H, B.J.G, K.C.G, R.H, R.S, W.S., D.W.S., M.J.H., U.C. and T.P‐P. have no competing interests. J.B. reports personal fees from Gilead Sciences, outside the submitted work. J.W.M. reports consulting agreements from Gilead Sciences, Inc., Merck, Xi'an Yufan Biotechnologies, Accelevir DX, ID Connect, and share options from ID Connect, Cocrystal Pharma, Inc., and Abound Bio, outside the submitted work.

## AUTHORS’ CONTRIBUTIONS

U.M.P. and J.W.M. led the study, developed the study design, analysis and interpretation. K.J.P developed the NGS and phenotyping assays and conducted data analysis and interpretation. A.L.H., E.K.H., B.J.G., K.C.G, and R.H. tested samples and conducted data analysis. R.S., W.S. and U.C. conducted bioinformatics analysis. D.W.S. and M.J.H. conducted statistical analysis. T.P‐P and J.M.B. conducted the ASPIRE study including study design, and the MTN‐020 Study Team conducted and implemented the ASPIRE study including data collection for this study. All co‐authors contributed to the writing and review of this manuscript.

## DISCLAIMER

The content is solely the responsibility of the authors and does not necessarily represent the official views of the National Institute of Allergy and Infectious Diseases or the National Institutes of Health.

## FUNDING INFORMATION

National Institute of Allergy and Infectious Diseases; Eunice Kennedy Shriver National Institute of Child Health and Human Development; National Institute of Mental Health; Grant/Award Number(s): UM1AI068633, UM1AI068615, and UM1AI106707.

## Members of the MTN‐020–ASPIRE Study Team

**Study Team Leadership**: Jared Baeten, University of Washington (Protocol Chair); Thesla Palanee‐Phillips, Wits Reproductive Health and HIV Institute (Protocol Co‐chair); Elizabeth Brown, Fred Hutchinson Cancer Research Center (Protocol Statistician); Lydia Soto‐Torres, US National Institute of Allergy and Infectious Diseases (Medical Officer); and Katie Schwartz, FHI 360 (Clinical Research Manager).

## Study Sites and Site Investigators of Record

*Malawi, Blantyre site (Johns Hopkins University, Queen Elizabeth Hospital)*: Bonus Makanani; *Malawi, Lilongwe site* (University of North Carolina, Chapel Hill): Francis Martinson; *South Africa, Cape Town site (University of Cape Town)*: Linda‐Gail Bekker; *South Africa, Durban, Botha's Hill, Chatsworth, Isipingo, Tongaat, Umkomaas, Verulam sites (South African Medical Research Council)*:  Vaneshree Govender; Samantha Siva, Zakir Gaffoor, Logashvari Naidoo, Arendevi Pather, and Nitesha Jeenarain; *South Africa, Durban, eThekwini site (Center for the AIDS Programme for Research in South Africa)*: Gonasagrie Nair; *South Africa, Johannesburg site (Wits RHI)*: Thesla Palanee‐Phillips; *Uganda, Kampala site (John Hopkins University, Makerere University)*: Flavia Matovu; *Zimbabwe, Chitungwiza, Seke South and Zengeza sites (University of Zimbabwe, University of California San Francisco)*: Nyaradzo Mgodi; *Zimbabwe, Harare, Spilhaus site (University of Zimbabwe, University of California San Francisco)*: Felix Mhlanga; Data management was provided by the Statistical Center for HIV/AIDS Research & Prevention (Fred Hutchinson Cancer Research Center, Seattle, WA) and site laboratory oversight was provided by the Microbicide Trials Network Laboratory Center (Pittsburgh, PA).

## Supporting information


**Table S1**: Ethics committee/review board approvals for the MTN‐020/ASPIRE protocol by clinical research site
