# Supplementary material for: HIV‐1 drug resistance among individuals who seroconverted in the ASPIRE dapivirine ring trial
Source: J Int AIDS Soc. 2021 Nov 11;24(11):e25833. doi: 10.1002/jia2.25833 (PMC8583424; doi:10.1002/jia2.25833)
Supplement: Supplementary file 1 — Table S1: Ethics committee/review board approvals for the MTN‐020/ASPIRE protocol by clinical research site [file JIA2-24-e25833-s001.pdf]

Table S1. Ethics Committee/Review Board Approvals for the MTN-020/ASPIRE Protocol by Clinical Research Site

Country	Clinical Research Site	Name of Ethics Committee/Review Board
**Malawi**	Blantyre	National Health Sciences Research Committee of Malawi<br>Johns Hopkins University Bloomberg School of Public Health Institutional Review Board
	Lilongwe	National Health Sciences Research Committee of Malawi<br>University of North Carolina at Chapel Hill Institutional Review Board
**South Africa**	Cape Town	University of Cape Town: Human Research Ethics Committee
	Durban (eThekwini, Botha's Hill, Chatsworth, Isipingo, Tongaat, Umkomaas, Verulum)	Biomedical Research Ethics Committee, University of KwaZulu-Natal
	Johannesburg	Wits Human Research Ethics Committee, University of Witwatersrand
**Uganda**	Kampala	Joint Clinical Research Centre Institutional Review Board<br>Johns Hopkins University School of Medicine Institutional Review Board
**Zimbabwe**	Chitungwiza – Seke South<br>Chitungwiza – Zengeza<br>Harare- Spilhaus	Medical Research Council of Zimbabwe<br>Committee on Human Research, University of California - San Francisco
